# Dermatofibrosarcome de Darier et Ferrand récidivant de la paroi abdominale: apport de la radiothérapie pré-opératoire (à propos d'un cas)

**DOI:** 10.11604/pamj.2022.41.234.22764

**Published:** 2022-03-22

**Authors:** Mohammed Amine Guerrouaz, Ayoub Kharkhach, Achraf Miry, Tijani El Harroudi, Ali Sbai, Loubna Mezouar

**Affiliations:** 1 Service de Radiothérapie, CHU Mohammed VI, Oujda, Maroc,; 2 Service de Chirurgie Générale, CHU Mohammed VI, Oujda, Maroc,; 3 Service d´Anatomo-Pathologie, CHU Mohammed VI, Oujda, Maroc

**Keywords:** Dermatofibosarcoma, local recurrence, MoHs surgery, case report, Dermatofibrosarcome, recidive locale, chirurgie de Mohs, cas clinique

## Abstract

Le dermatofibrosarcome est un cancer peu rencontré, représentant 0,01% de tous les cancers. Nous rapportons le cas d´une patiente âgée de 44 ans, admise chez nous pour une 5^e^ récidive d´un dermatofibrosarcome de Darier et Ferrand localement avancé, ayant progressé sous plusieurs lignes de chimiothérapie neoadjuvante, qui a nécessité une radiothérapie en urgence avec bonne réponse, ce qui nous a permis de réaliser une exérèse large de la tumeur avec de limites saines, la patiente est en rémission après un an de suivi. Le pronostic des DFS est généralement excellent. La chirurgie large et l´avènement de la chirurgie de Mohs ont permis d´améliorer le contrôle local. La place de radiothérapie est limité pour les tumeurs non resécables ou en cas de marges positives.

## Abstract (en)

Dermatofibrosarcoma is a rare cancer, accounting for 0.01% of all cancers. We here report the case of a 44-year-old female patient presenting with the 5^th^ recurrence of locally advanced Darier–Ferrand dermatofibrosarcoma, that progressed on many cycles of neoadjuvant therapy and required emergency radiotherapy with good response. This allowed to perform wide excision of the tumor with healthy limits. The patient had remission after 1 year of follow-up. Prognosis for patients with Darier-Ferrand dermatofibrosarcoma is generally excellent. Wide surgery and the advent of Mohs surgery have improved local control. The role of radiotherapy is limited for non-resectable tumors or positive margins.

## Introduction

Le dermatofibrosarcome est un cancer peu rencontré, il s´agit d´un sarcome d´origine fibroblastique de bas grade, il représente environ 0,01% de tous les cancers et 2 à 6% des sarcomes des tissus mous, aux états unis il a une incidence de 4,5 cas/1 million hab/1 an [1], il se caractérise par son agressivité locale, et par un potentiel métastatique faible. Le taux de récidive locale varie entre 10% à 60%, pourtant le taux de métastase régionales ou à distance est de seulement 1 à 4% [2,3]

## Patient et observation

Nous rapportons le cas d´une patiente âgée de 44 ans, sans antécédents pathologiques notable, admis au centre d´oncologie du CHU Mohammed VI d´Oujda pour une cinquième récidive d´un dermatofibrosarcome de Darier et Ferrand localement avancé après 4 exérèse dont l´examen initial à l´admission a objectivé une masse de la paroi abdominale sous ombilicale, rouge, ferme et douloureuse, de 10 x 10 cm. Le scanner thoraco-abdomino-pelvien a montré une volumineuse masse de la paroi antéro-médiane de l´abdomen mesurant 94 mm x 103 mm respectant le muscle grand droit de l´abdomen. Vu son caractère localement avancé, la décision prise en réunion de concertation pluridisciplinaire (RCP) était de la mettre sous un traitement néo-adjuvant pré-opératoire à base d´Imatinib. En fait la patiente a progressé après 3 cures d´Imatinib ce qui nous a poussé à changer de ligne vers une polychimiothérapie à base de Doxorubicine, Ifosfamide, Mesna (AIM), mais malheureusement la tumeur a été toujours en progression, une 3^e^ ligne a été instauré à base de Dacarbazine en monothérapie sans aucune réponse ni clinique ni radiologique.

**Figure 1:**
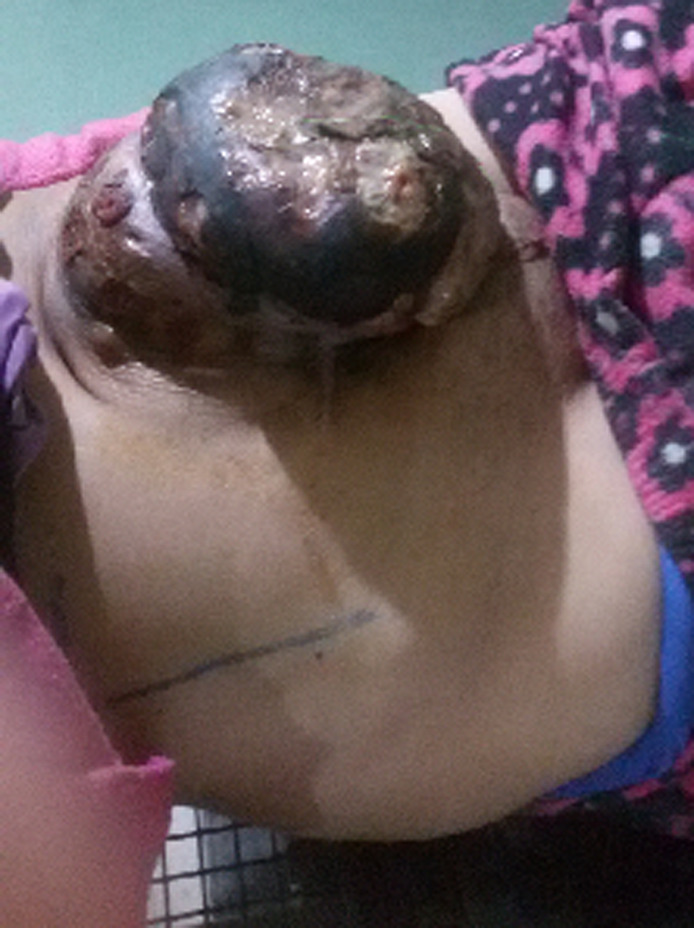
énorme masse tumorale au niveau de la paroi abdominale antérieure mesurant 16 cm de diamètre, ulcérée, saignante, et largement nécrosée

**Figure 2:**
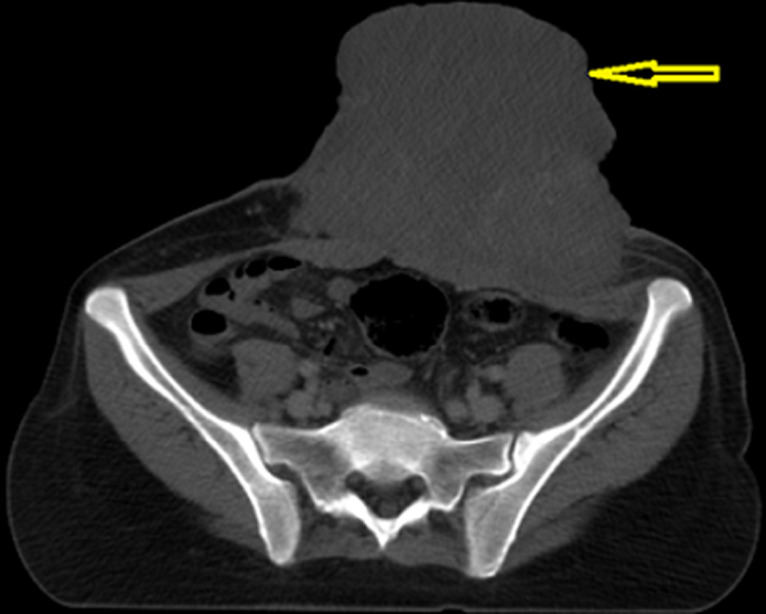
image scanographique objectivant la présence d´un énorme processus tumoral de la paroi abdominale antérieure infiltrant le muscle grand droit de l´abdomen gauche

**Figure 3:**
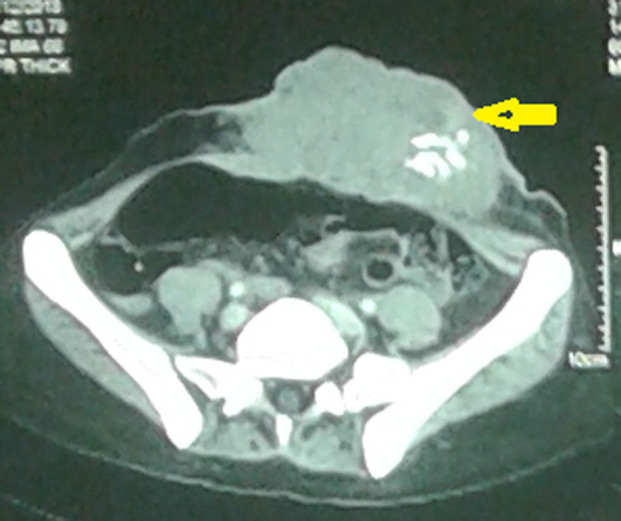
régression en taille du processus abdominal pariétal antérieur après fin de la radiothérapie avec présence de calcifications intra-tumoral

**Figure 4:**
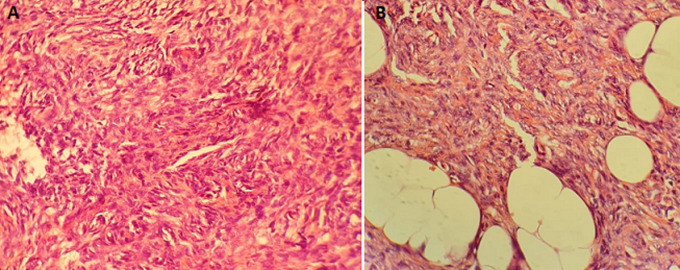
A, B) tumeur à cellule fusiforme évoquant un fibrosarcome de Darier et Ferrand

**Figure 5:**
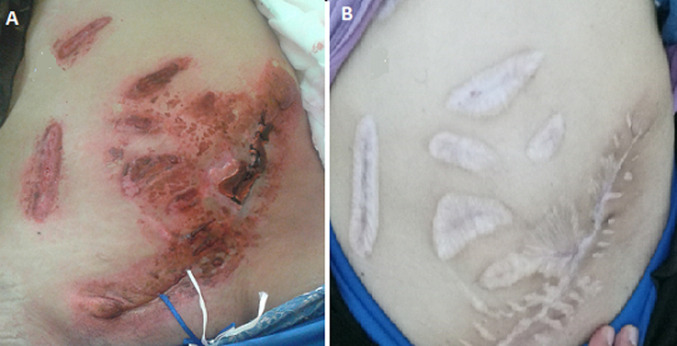
A) évolution clinique 2 mois après la résection; B) évolution clinique 1 an de suivi

**Figure 6:**
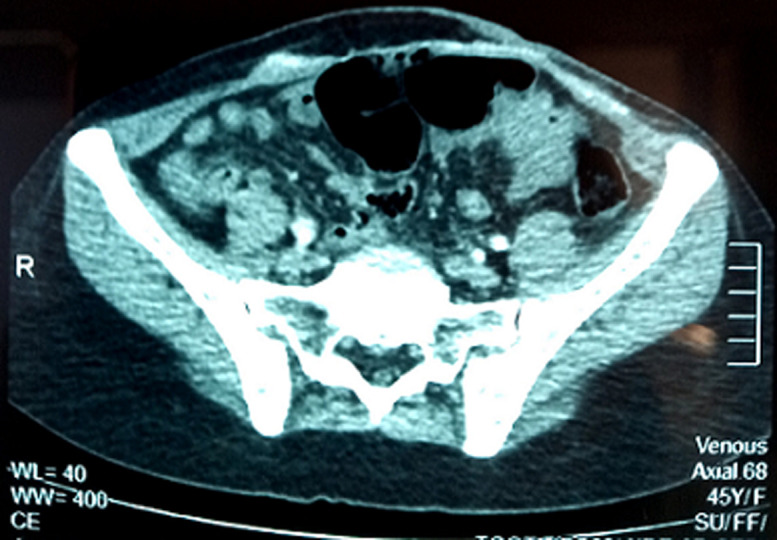
scanner un an après la chirurgie ne montrant aucun signe de récidive locale ni à distance

Elle a été adressée au service de radiothérapie pour une irradiation pré-opératoire, l´examen a objectivé une énorme masse tumorale au niveau de la région ombilicale mesurant 16 cm de diamètre, ulcérée, saignante, surinfectée et d´odeur fétide (Figure 1), biologiquement la patiente avait une anémie (une hémoglobine à 6g/dl) due à une hémorragie intra-tumorale. Un scanner TAP a été réalisé ayant objectivé une progression de la masse de la paroi antéro-médiane de l´abdomen infiltrant le muscle grand droit de l´abdomen gauche mesurant 152mm Vs 103mm (Figure 2). Vu que la tumeur était résistante au traitement médical et saignante, on a décidé de réaliser une radiothérapie pré-opératoire et hémostatique à la dose de 50 Gy en 25 fractions de 2 Gy avec une très bonne réponse clinique, puis on a évalué par un scanner un mois après: diminution de la taille du processus paroi abdominale antérieure (Figure 3). Ce qui a permi de faire une résection totale de la lésion dont l´examen anatomo-pathologique: tumeur à cellule fusiforme évoquant un fibrosarcome de Darrier et Ferrand de 12 cm de grand axe avec des limites saines de ≥ 1,5 cm pour la limite plus proche (Figure 4). L´évolution a été marquée par le contrôle local de la maladie un an après la chirurgie, aucun signe clinique ni radiologique n´a été objectivé (Figure 5, Figure 6).

## Discussion

Le dermarofibrosarcome (DFS) est un sarcome de faible grade dans la majorité des cas, son diagnostic est histologique par présence de cellules fusiformes, disposées de manière storiforme ou en forme de “chevrons” qui s´étend du derme à l´hypoderme. A l´immunohistomochimie, il est caractérisé la positivité de CD 34 (+) et la négativité du facteur XIIIa [4,5].

Jusqu´à l´heure actuelle le traitement de référence des DFS reste la chirurgie large, il est recommandé de respecter des marges de 2 à 4 cm voire 5 cm, ceci compte tenu des taux élevés de récidive locale [6]. La chirurgie de Mohs est aussi une option [7,8], l´avènement de cette dernière a permis d´augmenter le taux de marges de résection saines. En fait une étude comparant la résection large classique de DFS à la chirurgie de Mohs ayant porté sur 79 patients a montré que la résection large était associée à un taux de récidive de 13%, alors que la chirurgie de Mohs n'était suivie d'aucune récidive àprés 5 ans de suivi. Par contre une autre étude comparant les deux techniques n´a pas montré de différence en terme de contrôle local [9,10]. Enfin deux revues de la littérature ont montré un faible taux de récidive obtenu par la chirurgie de Mohs comparé à la chirurgie conventionnelle [11,12].

La radiothérapie n´est pas un traitement standard des sarcomes de Darier et Ferrand. Actuellement, il n'existe que peu de données pour soutenir son utilisation de routine. Le bénéfice apporté par la radiothérapie a été mis en évidence par peu d´études, elle permet d´obtenir un taux élevé de contrôle local dépassant les quatre-vingts pourcent et qui peut aller jusqu´à 98% à 5 ans [13-15]. Dans une étude prospective ayant recensé 53 patients, la chirurgie et la radiothérapie ont résulté dans un taux de contrôle local et une survie sans maladie à 10 ans de 93%, même pour les patients dont la chirurgie n´était pas complète avec résidu macroscopique [16]. Une autre étude a montré un taux de contrôle local à 86% après 10 ans de suivi [17]. D´après ces études il s´est avéré que les DFS sont radiosensibles et que la radiothérapie adjuvante peut être envisagée après une chirurgie incomplète ou une récidive si une re-exérèse n´est pas possible; en cas de marge saine aucun traitement adjuvant n´est nécessaire. Enfin chez les patients non candidats à une chirurgie, une radiothérapie exclusive peut constituer une option.

Mais ce ne sont par les seuls moyens thérapeutiques disponibles. Les DFS sont caractérisés par une translocation entre les chromosomes 17 et 22 t (17,22) avec sur-expression du récepteur PDGFRB qui est un récepteur exploitable qu´on peut cibler par l´Imatinib; en fait quelques études ont montré son intérêt, c´est un inhibiteur de la tyrosine kinase qui a prouvé son efficacité dans DFS localisés et métastatiques en cas de présence de la t (17,22), et par conséquence elle constitue une option en cas de tumeur non résécable, récidivant, ou métastatique et il est préférable de réaliser une étude cytogénétique à la recherche de la t (17,22) avant de l´instaurer [18]. En cas de maladie métastatique ayant progressé sous Imatinib on peut tenter une chimiothérapie par analogie aux sarcomes des tissu mous, il s´agit le plus souvent des protocoles à base d´une mono ou polyhimiothérapie par Doxotubicine, Ifosfamide, Epirubicine, Gemcitabine, Dacarbazine, Temozolomide, Vinorelbine, ou un inhibiteur de la tyrosine kinase (ITK) à base de Pazopanib [19].

## Conclusion

Le pronostic des DFS est généralement excellent. L´avènement de la chirurgie de Mohs a fait chuter le taux de récidive locale, elle permet d´obtenir un taux de guérison dans 98% des cas même en cas de DFS recidivant. Le taux de métastases à distance est de 4%; leur présence est associée à un mauvais pronostic. La radiothérapie généralement faite après une chirurgie permet d´assurer un contrôle local chez plus de 90% des cas. Compte tenu des premiers résultats encourageants obtenus avec l'imatinib dans les DFS, une amélioration du pronostic, même en cas de maladie métastatique, peut être envisagée.
